# Isolation of nontuberculous mycobacteria species from different water sources: a study of six hospitals in Tehran, Iran

**DOI:** 10.1186/s12866-022-02674-z

**Published:** 2022-10-29

**Authors:** Sina Moghaddam, Farshad Nojoomi, Arasb Dabbagh Moghaddam, Mojgan Mohammadimehr, Fatemeh Sakhaee, Morteza Masoumi, Seyed Davar Siadat, Abolfazl Fateh

**Affiliations:** 1 Department of Microbiology, School of Medicine, Aja University of Medical Sciences, Tehran, Iran; 2 Infectious Diseases Research Center, Aja University of Medical Sciences, Tehran, Iran; 3 Department of Mycobacteriology and Pulmonary Research, Pasteur Institute of Iran, Tehran, Iran; 4 Department of Public Health & Nutrition, Aja University of Medical Sciences, Tehran, Iran; 5 Department of Laboratory Sciences, Faculty of Paramedicine, Aja University of Medical Sciences, Tehran, Iran; 6 Microbiology Research Center (MRC), Pasteur Institute of Iran, Tehran, Iran

**Keywords:** Nontuberculous mycobacteria, Antibiotic resistance, Hospital drinking water

## Abstract

**Purpose:**

Nontuberculous mycobacteria (NTM) are ubiquitous bacteria that are naturally resistant to disinfectants and antibiotics and can colonize systems for supplying drinking water. Therefore, this study aimed to evaluate the prevalence of NTM in the drinking water of six hospitals in Tehran, Iran.

**Methods:**

Totally, 198 water samples were collected. Each water sample was filtered via a membrane filter with a pore size of 0.45 µm and then decontaminated by 0.005% cetylpyridinium chloride. The membrane filters were incubated on two Lowenstein-Jensen media at 25 °C and 37 °C for 8 weeks. The positive cultures were identified with phenotypic tests, and then NTM species were detected according to the *hsp65*, *rpoB*, and *16S rDNA* genes. Drug susceptibility testing (DST) was also carried out.

**Results:**

Overall, 76 (40.4%) of the isolates were slowly growing mycobacteria (SGM) and 112 (59.6%) of the ones were rapidly growing mycobacteria (RGM). The most common NTM were *Mycobacterium aurum*, *M. gordonae*, *M. phocaicum*, *M. mucogenicum*, *M. kansasii*, *M. simiae*, *M. gadium*, *M. lentiflavum*, *M. fortuitum*, and *M. porcinum*. Among these 188 samples, NTM ranged from 1 to > 300 colony-forming unit (CFU) /500 mL, with a median of 182 CFU/500 mL. In the infectious department of all hospitals, the amount of CFU was higher than in other parts of the hospitals. The DST findings in this study indicated the diversity of resistance to different drugs. Among RGM, *M. mucogenicum* was the most susceptible isolate; however, *M. fortuitum* showed a different resistance pattern. Also, among SGM isolates, *M. kansasii* and *M. simiae*, the diversity of DST indicated.

**Conclusions:**

The current study showed NTM strains could be an important component of hospital water supplies and a possible source of nosocomial infections according to the CFU reported in this study. The obtained findings also help clarify the dynamics of NTM variety and distribution in the water systems of hospitals in the research area.

**Supplementary Information:**

The online version contains supplementary material available at 10.1186/s12866-022-02674-z.

## Introduction

Due to their high cost, challenging treatment, and rising prevalence, infections caused by nontuberculous mycobacteria (NTM) have attracted more interest in the medical and engineering industries during the past few decades [1, 2]. The NTM, also known as “environmental mycobacteria”, are common in soil, freshwater sources, public water supplies, and household dust and comprise numerous opportunistic diseases [3]. It is believed that most NTM infections result from human–environment interactions, such as the inhalation of contaminated particles and aerosols [4].

As bacteria live in the environment, changes in climatic circumstances are likely to have an effect on NTM. When the industrial revolution started, more greenhouse gases, such as carbon dioxide and methane, were released into the atmosphere. This has caused average temperatures on land and in the ocean to rise by about 0.2 °C per decade [5]. Both rising levels of carbon dioxide in the atmosphere and average temperatures worldwide have ripple effects. Some of these changes are higher humidity, heavier rain and drought, more frequent and/or bigger natural disasters, longer warm seasons, and changes in the sea level [5]. Coastal areas are especially affected by climate change, and in the United States, many of these areas are the same places where most NTM infections occur, such as Hawaii, Louisiana, California, and Florida [3, 6].

Some critical characteristics that determine NTM survival should be known to speculate on how the likelihood of NTM infections might alter in future circumstances. The NTM have a lipid-rich outer membrane consisting of mycolic acids that provides them with several qualities that help them stay alive, such as being water-repellent, impermeable, and slow-growing [3]. The NTM, due to their hydrophobicity, have a tendency to develop biofilms on surfaces, such as water pipe walls. However, due to their negative surface charge, NTM can also adsorb into these surfaces [3, 7]. They have been observed to thrive in generally harsh environments, such as acidic, heated, oligotrophic, and microaerobic conditions [4, 8].

More than 200 NTM species are currently identified, and they can be grouped into two main categories, including slowly growing mycobacteria (SGM) and rapidly growing mycobacteria (RGM) [9]. The species with the greatest clinically relevance differ by region across the world and have altered over time [10]. Nevertheless, a number of SGM (*Mycobacterium avium*, *M. xenopi*, *M. malmoense*, *M. intracellulare*, *M. marinum*, and *M. kansasii*) and RGM (*M. chelonae*, *M. abscessus,* and *M. fortuitum*) species are today of considerable clinical interest. *M. gordonae*, an additional significant slow-growing species, is common in drinking water supply but less frequently associated with disease [4].

As the number of cases of NTM disease keeps going up, global climate change speeds up, and population dynamics change, it is necessary to know as soon as possible how the risk of NTM infections might change in the coming decades [3]. In both industrialized and developing nations, NTM are among the main causes of opportunistic nosocomial infections in a variety of therapeutic settings; however, little is known about their isolation and detection in Iranian hospitals. Therefore, this study aimed to determine how common NTM was in Tehran’s hospital aquatic systems in Iran.

## Materials and methods

### Sample collection

Within January 2022 and July 2022, a total of 198 water samples were taken from various sites of teaching hospitals in the Tehran, Iran. All six hospitals had similar departments, which were including emergency, men and Women internal, intensive care unit (ICU), coronary care unit (CCU), infectious, operating room, laboratory, dentistry, dialysis, and heart surgery. All water samples were chlorinated drinking water. The samples included tap water from the emergency department (*n* = 12), women's internal medicine (*n* = 6), men's internal medicine (*n* = 6), women's surgery center (*n* = 18), men's surgery center (*n* = 6), ICU (*n* = 24), CCU (*n* = 12), operating room (*n* = 12), laboratory (*n* = 10), dentistry unit water (*n* = 14), department of Infectious diseases (*n* = 24), hemodialysis center fluid (*n* = 36), angiography department (*n* = 14), and heater-cooler devices (*n* = 4). Each sample was prepared in a sterile glass container with a volume of around 500 ml (mL), brought to the lab in an icebox, and examined within 24 h.

### Sample decontamination and culture

The processing and decontamination of all samples were performed as previously described [11]. Briefly, each water sample (500 mL) was filtered via a membrane filter with a pore size of 0.45 µm (Millipore, Bedford, United States of America) and then decontaminated by 0.005% cetylpyridinium chloride for 30 min at room temperature. The membranes were washed with sterile distilled water to remove excess residual decontamination substances and then were incubated on two Lowenstein-Jensen media at 25 °C and 37 °C for 8 weeks. Colony morphology, growth rate, and pigmentation were all monitored twice a week in the cultures. After growing, the presence of acid-fast bacilli (AFB) in colonies was determined using the Ziehl–Neelsen staining. All positive samples for AFB were tested for phenotypic tests, including urease production, nitrate reduction, salt tolerance, arylsulfatase, tellurite reduction, niacin accumulation, tween-80 hydrolysistellurite, and semiquantitative catalase production, according to the Centers for Disease Control and Prevention procedures [12].

### DNA extraction and molecular identification

Total deoxyribonucleic acid (DNA) was extracted using a DNA kit (SinaCloneBioScience, Tehran, Iran). Two specific primers, TB11 (5'-ACCAACGATGGTGTGTCCAT-3') and TB12 (5'-CTTGTC GAACCGCA-TACCCT-3'), were used to amplify a 441-bp fragment of the heat shock protein 65 (*hsp65*) gene. The polymerase chain reaction (PCR) products were then sequenced [13, 14]. According to Adékambi and colleagues, two primers, F-(5'-GGCAAGGTCACCCCGAAGGG-3') and R-(5'-AGCGGCTGCTGGGTGATCATC-3'), were used to amplify and sequence a 750-bp specific region of the *rpoB* gene [15, 16]. In addition, a 1500-bp fragment of the *16S rDNA* gene was also amplified from the isolates using the previously published primers pA (5'-AGAGTTTGATCCTGGCTCAG-3') and pI (5'-TGCACACAGGCCACAAGGGA-3') [17, 18]. The PCR reaction for the *hsp65*, *rpoB*, and *16S rDNA* genes was carried out in a total volume of 25 mL containing 0.125 mM deoxynucleotide triphosphates, Taq DNA Polymerase (1.5 U), 10 pM of each primer, 2 mM MgCl2, and 50 ng of DNA. PCR for the *hsp65* gene was performed as follows: 94 °C for 4 min, 30 cycles of 94 °C for 40 s, 60 °C for 35 s, 72 °C for 60 s and a final extension at 72 °C for 10 min. PCR for the *rpoB* gene was performed as follows: 94 °C for 6 min, 40 cycles of 94 °C for 45 s, 58 °C for 40 s, 72 °C for 45 s, and a final extension at 72 °C for 10 min. PCR for *16S rDNA* was conducted as follows: 95 °C for 5 min, 35 cycles of 95 °C for 45 s, 62 °C for 45 s, 72 °C for 45 s, and final extension at 72 °C for 10 min.

Moreover, PCR products were purified with the AccuPrep® PCR purification kit (Bioneer, Seoul, South Korea). The PCR products were sequenced using an ABI Automated Sequencer (Applied Biosystems, Foster City, California, United States) and the raw data were evaluated with the MEGA version 11.0 program (https://www.megasoftware.net/).

### Drug susceptibility testing (DST)

The broth microdilution method was used to determine each drug's minimum inhibitory concentrations (MICs), which were then interpreted in accordance with the Clinical and Laboratory Standards Institute (CLSI) recommendations [19]. The examined drugs included rifampicin, ethambutol, isoniazid, ofloxacin, capreomycin, ethionamide, streptomycin, ciprofloxacin, amikacin, levofloxacin, ciprofloxacin, clarithromycin, imipenem, trimethoprim sulfamethoxazole, vancomycin, doxycycline, minocycline, and cefoxitin. The serial twofold dilutions of medicines ranging from 0.06 to 512 mg/L were prepared and added to Middlebrook 7H9 Supplement with the addition of 5% albumin-dextrose-catalase (ADC). All cultures were incubated aerobically at 25 °C and 37 °C following inoculation. The growth rate was assessed on the 3^rd^ and 7^th^ days for RGM and weekly for SGM up to 4 weeks. The MIC was the lowest concentration of antimicrobial drugs that inhibits observable growth. According to CLSI recommendations, susceptible, moderately susceptible, and resistant breakpoints were assigned.

## Results

### NTM species identification by phenotypic tests

The NTM strains were divided into two groups based on their growth rates. Overall, 76 (40.4%) of the isolates were SGM and 112 (59.6%) of the ones were RGM. Moreover, 38 (15.9%) and 80 (42.6%) isolates were photochromogenic and scotochromogenic bacteria, respectively. Totally, 62 (33.0%) and 126 (67.0%) of the isolates were grown at 37 °C and 25 °C, respectively. The most common strain identified by biochemical tests was *M. gordonae* (24 isolates), followed by *M. kansasii* (18 isolates), *M. simiae* (18 isolates), *M. fortuitum* (12 isolates), and *M. chelonae* (4 isolates). Biochemical tests could only identify 76 (40.4%) of the 188 isolates investigated in this study. The rest of the isolates remained unidentified. Totally, 128 (64.6%) of 198 water samples were positive for NTM species. Additionally, 59 (29.8%) samples were negative, and the remaining samples 11 (5.6%) were contaminated (Table 1).

**Table 1:** Distribution of water samples from hospitals in Tehran, Iran

Hospital (No. of samples)	Water samples (*n* = 198)		
	Positive	Negative	Contaminated
No. 1 (*n* = 56)	52 (92.8%)	3 (5.4%)	1 (1.8%)
No. 2 (*n* = 56)	36 (64.3%)	15 (26.8%)	5 (0.9%)
No. 3 (*n* = 22)	10 (45.5%)	10 (45.5%)	2 (9.0%)
No. 4 (*n* = 22)	8 (36.4%)	14 (63.6%)	0 (0.0%)
No. 5 (*n* = 22)	8 (36.4%)	12 (54.5%)	2 (9.1%)
No. 6 (*n* = 20)	14 (70.0%)	5 (25.0%)	1 (5.0%)

### NTM species detection by molecular tests

Table 2 shows the distribution of water samples in different sources from six hospitals in Tehran province. Based on molecular tests (*hsp65*, *rpoB*, and *16S rDNA* genes), NTM isolates included *M. aurum* (28 isolates), *M. gordonae* (24 isolates), *M. phocaicum* (20 isolates), *M. mucogenicum* (20 isolates), *M. kansasii* (18 isolates), *M. simiae* (18 isolates), *M. gadium* (16 isolates), *M. lentiflavum* (12 isolates), *M. fortuitum* (12 isolates), *M. porcinum* (8 isolates), *M. chelonae* (4 isolates), *M. florentinum* (4 isolates), *M. moriokaense* (2 isolates), and *M. novocastrense* (2 isolates). Interestingly, in some samples, more than one mycobacterial species was isolated; for example, three strains, including *M. mucogenicum*, *M. gordonae*, and *M. novocastrense*, were isolated from one of the samples.

**Table 2:** Results of NTM identification by molecular test in different temperature

Incubated at 25 °C	Water collection sources													
Mycobacterium species(RGM or SGM)	Emergency (*n* = 12)	Women internal (*n* = 6)	Men internal (*n* = 6)	Men surgery (*n* = 6)	Women surgery (*n* = 18)	ICU (*n* = 24)	CCU (*n* = 12)	Infectious (*n* = 24)	Operating room (*n* = 12)	Laboratory (*n* = 10)	Dentistry (*n* = 14)	Dialysis (*n* = 36)	Heart surgery (*n* = 18)	Total (*n* = 198)
*M. aurum* (R)	2	0	2	0	6	2	0	2	0	0	8	0	6	28
*M. gordonae* (S)	0	4	2	0	0	2	0	10	2	2	0	2	0	24
*M. gadium* (R)	2	0	0	0	2	8	4	0	0	0	0	0	0	16
*M. phocaicum* (R)	0	0	0	0	4	2	2	0	0	0	4	2	4	18
*M. mucogenicum* (R)	0	0	0	2	2	2	0	4	2	2	0	2	0	16
*M. chelonae* (R)	0	0	0	0	0	0	0	0	0	0	0	2	2	4
*M. moriokaense* (R)	0	0	0	2	0	0	0	0	0	0	0	0	0	2
*M. novocastrense* (R)	0	0	0	0	0	0	0	2	0	0	0	0	0	2
*M. lentiflavum* (S)	4	0	0	0	0	4	0	0	2	0	0	2	0	12
*M. florentinum* (S)	2	0	0	0	0	0	0	2	0	0	0	0	0	4
Incubated at 37 °C														
*M. phocaicum* (R)	0	0	0	0	0	0	0	0	2	0	0	0	0	2
*M. mucogenicum* (R)	0	0	0	0	0	0	0	0	0	2	0	0	2	4
*M. porcinum* (R)	0	0	0	0	0	0	0	0	0	0	6	0	2	8
*M. kansasii* (S)	0	4	0	0	6	4	0	2	2	0	0	0	0	18
*M. fortuitum* (R)	0	0	0	0	2	0	0	6	0	0	0	0	4	12
*M. simiae* (S)	0	0	0	4	0	2	0	8	0	0	0	2	2	18
Total positive samples	10	8	4	8	22	26	6	36	10	6	18	12	22	188

SGM and RGM strains have been isolated in all hospitals. Colony-forming unit (CFU) were enumerated in all samples from which NTM were isolated. Among these 188 samples, NTM ranged from 1 to > 300 CFU/500 mL, with a median of 182 CFU/500 mL. In the infectious department of all hospitals, the amount of CFU was higher than in other parts of the hospitals (Supplementary Table 1).

### Results of DST for most common NTM

Table 3 shows the MICs, the MIC_50_ and MIC_90_, and the interpretation of antimicrobial drugs against NTM isolates. The most resistant profile among the *M. aurum* isolates were for cefoxitin (57.1%), imipenem (42.6%), ciprofloxacin (42.6%), and moxifloxacin (39.1%), respectively. The highest resistance among *M. phocaicum* isolates was related to ciprofloxacin (80.0%), doxycycline (50.0%), and tobramycin (50.0%).

**Table 3:** Antimycobacterial susceptibility testing results for environmental isolates

Bacteria (no.) and antimicrobial agent		MIC (µg/ml)		No. (%) of isolates		
	Range	50%	90%	Susceptible	Intermediate	Resistant
*M. aurum* (*n* = 28)						
Clarithromycin	0.5—≥ 16	0.5	≥ 8	15 (53.8%)	2 (7.1%)	9 (39.1%)
Tobramycin	2 – ≤ 2–4	2	4	28 (100.0%)	0 (0.0%)	0 (0.0%)
Amikacin	≤ 1– ≤ 1–2	≤ 1	2	21 (75.0%)	0 (0.0%)	7 (25.2%)
Doxycycline	≤ 0.25 – ≤ 0.25–0.50	0.25	0.50	17 (60.7%)	1 (3.6%)	10 (35.7%)
Cefoxitin	≤ 4 – ≤ 2–8	4	8	8 (28.6%)	4 (14.3%)	16 (57.1%)
Trimethoprim/sulfamethoxazole	≤ 0.25 – 4	0.25	4	25 (89.3%)	1 (3.6%)	2 (7.1%)
Linezolid	≤ 1– ≤ 2	≤ 1	1	27 (96.4%)	0 (0.0%)	1 (3.6%)
Ciprofloxacin	≤ 0.12– ≤ 0.5	≤ 0.12	0.25	15 (53.8%)	1 (3.6%)	12 (42.6%)
Meropenem	≤ 1– ≤ 2	≤ 1	1	20 (71.4%)	0 (0.0%)	8 (28.6%)
Imipenem	≤ 1– ≤ 2	≤ 1	1	15 (53.8%)	1 (3.6%)	12 (42.6%)
Moxifloxacin	≤ 0.25– ≤ 0.25	0.06	≤ 0.25	15 (53.8%)	2 (7.1%)	11 (39.1%)
*M. phocaicum* (n = 20)						
Clarithromycin	≤ 2—≥ 8	≤ 2	≥ 8	18 (90.0%)	0 (0.0%)	0 (10.0%)
Tobramycin	≤ 4–8 – ≥ 32	4	8	9 (45.0%)	1 (5.0%)	10 (50.0%)
Amikacin	≤ 16–32 – ≥ 64	16	32	19 (95.0%)	0 (0.0%)	1 (5.0%)
Doxycycline	≤ 1–8 – ≥ 16	4	16	8 (40.0%)	2 (10.0%)	10 (50.0%)
Cefoxitin	≤ 16–32 – ≥ 128	16	64	17 (85.0%)	0 (0.0%)	3 (25.0%)
Trimethoprim/sulfamethoxazole	≤ 1—≥ 4	1	4	19 (95.0%)	0 (0.0%)	1 (5.0%)
Linezolid	≤ 8–16 – ≥ 32	8	32	18 (90.0%)	0 (0.0%)	0 (10.0%)
Ciprofloxacin	≤ 1–2 – ≥ 4	1	4	3 (15.1%)	1 (5.0%)	16 (80.0%)
Meropenem	≤ 4 – 8	2	4	15 (75.0%)	1 (5.0%)	4 (20.0%)
Imipenem	≤ 4–8 – ≥ 16	4	8	17 (85.0%)	0 (0.0%)	3 (25.0%)
Moxifloxacin	≤ 1–2 – ≥ 4	1	4	19 (95.0%)	0 (0.0%)	1 (5.0%)
*M. mucogenicum* (n = 16)						
Clarithromycin	≤ 2–4—≥ 8	2	8	15 (93.8%)	0 (0.0%)	1 (6.2%)
Tobramycin	≤ 4–8 – ≥ 16	4	16	14 (87.5%)	1 (6.2%)	1 (6.2%)
Amikacin	0.5 –64	0.5	32	16 (100.0%)	0 (0.0%)	0 (0.0%)
Doxycycline	0.25 –32	0.25	16	11 (68.8%)	1 (6.2%)	4 (25.0%)
Cefoxitin	≤ 16–32 – ≥ 128	8	64	15 (93.8%)	0 (0.0%)	1 (6.2%)
Trimethoprim/sulfamethoxazole	≤ 1—4	1	4	15 (93.8%)	1 (6.2%)	0 (0.0%)
Linezolid	1–4 – 128	1	64	15 (93.8%)	0 (0.0%)	1 (6.2%)
Ciprofloxacin	≤ 1– ≥ 128	1	64	14 (87.5%)	0 (0.0%)	2 (12.5%)
Meropenem	≤ 4 – ≥ 16	4	8	12 (75.0%)	0 (0.0%)	4 (25.0%)
Imipenem	≤ 1 – ≥ 128	1	64	15 (93.8%)	0 (0.0%)	1 (6.2%)
Moxifloxacin	≤ 1–2 – ≥ 4	1	4	15 (93.8%)	1 (6.2%)	0 (0.0%)
*M. fortuitum* (*n* = 12)						
Clarithromycin	0.06–64	2	8	8 (66.7%)	0 (0.0%)	4 (33.3%)
Tobramycin	0.25–32	2	4	11 (91.7%)	0 (0.0%)	1 (8.3%)
Amikacin	0.125–128	1	8	11 (91.7%)	0 (0.0%)	1 (8.3%)
Doxycycline	0.25–128	8	32	2 (16.7%)	0 (0.0%)	10 (83.8%)
Cefoxitin	2–256	32	64	4 (33.3%)	1 (8.3%)	7 (58.4%)
Trimethoprim/sulfamethoxazole	1–32	2	8	11 (91.7%)	1 (8.3%)	0 (0.0%)
Linezolid	0.25–64	2	32	8 (66.7%)	1 (8.3%)	3 (25.0%)
Ciprofloxacin	0.06–16	0.25	8	5 (41.7%)	1 (8.3%)	6 (50.0%)
Meropenem	1–128	4	32	6 (50.0%)	1 (8.3%)	4 (33.3%)
Imipenem	0.06–64	1	8	5 (41.7%)	1 (8.3%)	6 (50.0%)
Moxifloxacin	0.06–64	0.125	16	7 (58.4%)	0 (0.0%)	5 (41.7%)
*M. kansasii* (*n* = 18)						
Isoniazid	0.25–16	2	4	18 (100.0%)	0 (0.0%)	0 (0.0%)
Rifampicin	0.125–16	2	8	5 (27.8%)	1 (5.5%)	12 (66.7%)
Ethambutol	0.25–64	2	4	18 (100.0%)	0 (0.0%)	0 (0.0%)
Clarithromycin	0.125–32	0.125	2	18 (100.0%)	0 (0.0%)	0 (0.0%)
Moxifloxacin	0.125–2	0.125	1	18 (100.0%)	0 (0.0%)	0 (0.0%)
Linezolid	0.125–2	0.125	1	18 (100.0%)	0 (0.0%)	0 (0.0%)
Ciprofloxacin	0.25–16	1	4	5 (22.2%)	2 (11.1%)	12 (66.7%)
*M. simiae* (*n* = 18)						
Isoniazid	16–128	32	64	0 (0.0%)	0 (0.0%)	18 (100.0%)
Rifampicin	0.5–128	4	64	0 (0.0%)	0 (0.0%)	18 (100.0%)
Ethambutol	4–64	8	32	0 (0.0%)	0 (0.0%)	18 (100.0%)
Streptomycin	2–64	16	32	0 (0.0%)	0 (0.0%)	18 (100.0%)
Amikacin	0.06–64	2	16	16 (89.0%)	1 (5.5%)	1 (5.5%)
Ofloxacin	0.25–64	4	32	17 (94.5%)	1 (5.5%)	0 (0.0%)
Ciprofloxacin	0.25–64	4	32	16 (89.0%)	0 (0.0%)	2 (11.0%)
Trimethoprim/sulfamethoxazole	16–512	32	256	0 (0.0%)	0 (0.0%)	18 (100.0%)
Clarithromycin	32–128	32	64	0 (0.0%)	0 (0.0%)	18 (100.0%)

*MIC* Minimum inhibitory concentrations

The highest resistance patterns among the *M. mucogenicum* were for doxycycline (25.0%) and meropenem (25.0%) and among *M. fortuitum* isolates was for doxycycline (83.8%), cefoxitin (58.4%), and imipenem (50.5%).

Among the 18 isolates of *M. kansasii*, 66.7% isolates were resistant to ciprofloxacin and rifampicin and all *M. simiae* strains were resistant to isoniazid, rifampicin, ethambutol, trimethoprim/sulfamethoxazole, clarithromycin, and streptomycin.

## Discussion

The NTM are one type of bacteria that are thought to be opportunistic plumbing pathogens. Direct and indirect exposure to tap water in healthcare settings has been linked to the outbreaks of healthcare-associated diseases and even mortality. Consequently, water management systems are crucial components of both facility management and infection control strategies [20, 21].

In this study, both phenotypic and molecular methods were used to find and identify NTM species in water samples from six hospitals. The AFB was observed in 128 (64.6%) of the samples, 59.6% and 40.4% of which were RGM and SGM strains, respectively. The recovery rate in this study is higher than what other studies of hospital water have shown in Iran [22, 23]. The prevalence of RGM and SGM isolates in hospital water samples in Iran ranged from 42.2%-67.5% and 32.5%-57.7%, respectively. It seems that the frequency of RGM is higher than SGM in Iran. It should be noted that NTMs geographic distribution differs by region [23, 24].

The most common SGM isolates were *M. lentiflavum* (84.7%), *M. avium* complex (2.8%-56.4%), and *M. gordonae* (2.8%-56.3%); nevertheless, the most common RGM isolates were *M. fortuitum* (2.9%-44.2%), *M. chelonae* (8%-36.8%), and *M. mucogenicum* (8%-25.6%) [24].

The present study showed that the most prevalent NTM isolated from hospital water were *M. aurum*, *M. gordonae*, *M. phocaicum*, *M. kansasii*, *M. simiae*, *M. gadium*, *M. mucogenicum*, *M. lentiflavum*, and *M. fortuitum*. Although some mycobacteria, such as *M. lentiflavum* and *M. gordonae*, are thought to be non-pathogenic, there have been reports of infections produced by these bacteria [16, 25].

Despite the fact that a large percentage of NTM species have been discovered from drinking water distribution systems in Finland (between 30–80%), France (72%), Australia (62%), and Germany (57%), the chemical and microbiological properties of these samples have not been published [26, 27].

It has been discovered that water meters, which are often located at the most distant point of the distribution system, harbor biofilms that are infected with mycobacteria [28]. In this context, the species *M. gordonae*, *M. aurum*, *M. terrae*, *M. avium*, *M. shimoidei*, *M. haemophilum*, *M. marinum*, and *M. intracellulare* have been extracted from water meter biofilm samples.

Chlorination is the most commonly used disinfection procedure, and the most chlorine-releasing chemicals are mycobacteriocidal. However, mycobacteria are quite resistant to most disinfectants due to their complex cell wall [29]. For example, the data showed that *M. chelonae* and *M. fortuitum* were more resistant; nevertheless, *M. aurum* seemed to be the most sensitive mycobacterial species to chlorine. However, the chlorine resistance rates of *M. aurum* and *M. gordonae* are 100 and 330 times greater than *Escherichia coli*, respectively [30].

Some of the NTM species isolated in this study including *M. mucogenicum*, *M. kansasii*, *M. simiae*, and *M. fortuitum*, were potentially pathogenic to humans. It has been reported that several NTM species have been correlated with waterborne transmission of human illness, including *M fortuitum*, *M. marinum*, *M. ulcerans*, *M. chelonae*, *M. abscessus*, *M kansasii*, *M ulcerans*, *M. szulgai*, *M. phocaicum*, and *M. simiae*. The number of potentially harmful mycobacterial species whose transmission route is related to water has increased. This finding is partly due to exposures in workplaces and institutions that led to respiratory diseases that were previously undiagnosed. Undoubtedly, as epidemiology and surveillance methods advance, more species will appear as water-related illnesses [23, 26, 31].

In the current study, the highest amount of CFU (> 300 CFU/500 mL) among NTM strains were detected in the department of Infectious diseases, women's surgery center (NTM ranged from 50 to > 300 CFU/500 mL), heart surgery (NTM ranged from 10 to > 300 CFU/500 mL), and ICU (NTM ranged from 5 to > 300 CFU/500 mL), respectively. Infections caused by NTM that are unrelated to cardiothoracic surgery are not generally considered to be a public health concern [32]. However, to prevent the further spread of the disease, research should be conducted on the healthcare-related transmission of NTM infections and healthcare-related hypersensitive lung disease from indoor stagnant water sources and surgical operations involving exposure to NTM-contaminated liquid. On account of reports of infections caused by other mycobacterial species related to *M. chimaera*, a general recommendation is to avoid using tap water or ice derived from tap water when manipulating intravenous catheters and endoscopes or using them in the operating room, particularly during cardiac surgery and during mammoplasty [33, 34].

Hemodialysis patients face a serious health risk when hemodialysis fluid is contaminated with whole bacteria cells or their pieces. In this study, from 36 hemodialysis water samples, 12 NTM species (ranged from 1 to > 300 CFU/500 mL), including *M. gordonae*, *M. phocaicum*, *M. mucogenicum*, *M. chelonae*, *M. lentiflavum*, and *M. simiae*, were isolated. High rates of contamination in dialysis water fluid were observed in various medical settings, which is consistent with the findings of the current study. Nine hospitals in Japan's hemodialysis centers reported significant dialysate contamination rates. Of the 40 dialysate samples that were examined, 42.5% had bacterial counts that were higher than 2000 CFU/mL, which was higher than the recommended level [35]. In a related study carried out in Brazil, 19 hemodialysis machines were used to identify 11 mycobacterial species, including *M. lentiflavum*, *M. gordonae*, *M. kansasii*, and *M. gastri* [36]. Furthermore, in a recent Iranian study, several NTM species, including *M. fortuitum*, *M. gordonae*, *M. mucogenicum*, *M. abscessus*, *M. chelonae*, *M. simiae*, and *M. kansasii* were detected in 65 samples originating from the hemodialysis distribution system [37].

The DST finding in this study indicated the diversity of resistant to different drugs. Among RGM, *M. mucogenicum* was the most susceptible isolate, however, *M. fortuitum*, *M. aurum,* and *M. phocaicum* showed a different resistance patterns. Additionally, among SGM isolates, *M. kansasii* and *M. simiae*, the diversity of DST indicated. This result was in consistent with the results of a study on pathogenic NTM in patients with pulmonary disease [18]. Since the individuals who are generally susceptible to NTMs have an underlying disease, it can be problematic for these individuals to be infected with NTMs with various types of drug resistance. However, the evaluation of drug resistance in NTM strains isolated from the environment can be effective in infection control.

The present study has some limitations. The sampling duration was short (only in summer) and did not encompass the entire seasonal variation that may occur in Iran. In addition, this study did not examine the mechanism underlying the resistance of NTM to the individual tested antimicrobial agents.

In conclusion, this report was a contribution toward the improvement of understanding regarding the presence of NTM in the water of six hospitals in Tehran. Further studies are required to determine the presence, abundance, and infection ability of NTM in plumbing systems from hospitals that host vulnerable populations, such as immunocompromised, children, and elderly cases. Furthermore, in such facilities, education and more advanced treatment techniques should be employed to inactivate NTM and other opportunistic waterborne diseases.

## Supplementary Information

**Additional file 1:** **Supplementary Table 1.** the frequency of NTM isolates and CFU in six-hospitals.

## Data Availability

The data used to support the findings of this study are included within the article and supplementary file. The nucleotide sequence data are available in the GenBank databases under the accession numbers OP580654 to OP580841 for the *16S rDNA*, OP598205 to OP598392 for hsp68, and OP598393 to OP598580 for *rpoB* genes.
