# Supplementary material for: Isolation of nontuberculous mycobacteria species from different water sources: a study of six hospitals in Tehran, Iran
Source: BMC Microbiol. 2022 Oct 29;22:261. doi: 10.1186/s12866-022-02674-z (PMC9617398; doi:10.1186/s12866-022-02674-z)
Supplement: Supplementary file 1 — Additional file 1: Supplementary Table 1. the frequency of NTM isolates and CFU in six-hospitals. [file 12866_2022_2674_MOESM1_ESM.docx]

**Supplementary Table 1:** the frequency of NTM isolates and CFU in six-hospitals

| **Row** | **Hospital number** | **Sampling location** | **Isolated strain- Incubated at 25°C** | **CFU 500 ml^-1^** |
| --- | --- | --- | --- | --- |
| 1 | No. 1 | Emergency1/ Tap water | *M. aurum* | 100 |
| 2 |  | Emergency2/ Tap water | *M. aurum* | 50 |
| 3 |  | Women internal1/ Tap water | *M. gordonae* | >300 |
| 4 |  | Women internal2/ Tap water | *M. gordonae* | >300 |
| 5 |  | Women internal1/ Tap water | *M. gordonae* | >300 |
| 6 |  | Women internal2/ Tap water | *M. gordonae* | >300 |
| 7 |  | ICU1/ Tap water | *M. gadium* | >300 |
| 8 |  | ICU2/ Tap water | *M. gadium* | >300 |
| 9 |  | Women surgery1/Tap water | *M. gadium* | >300 |
| 10 |  | Women surgery2/Tap water | *M. gadium* | >300 |
| 11 |  | Women surgery1/Tap water | *M. phocaicum*  *M. aurum* | 20  >300 |
| 12 |  | Women surgery2/Tap water | *M. phocaicum*  *M. aurum* | 50  200 |
| 13 |  | CCU1/ Tap water | *M. phocaicum* | >300 |
| 14 |  | CCU2/ Tap water | *M. phocaicum* | 100 |
| 15 |  | Men internal1/ Tap water | *M. aurum* | >300 |
| 16 |  | Men internal2/ Tap water | *M. aurum* | >300 |
| 17 |  | Women surgery1/ Tap water | *M. aurum* | >300 |
| 18 |  | Women surgery2/ Tap water | *M. aurum* | >300 |
| 19 |  | Women surgery1/ Tap water | *M. aurum* | >300 |
| 20 |  | Women surgery2/ Tap water | *M. aurum* | >300 |
| 21 |  | ICU1/ Tap Water | *M. gadium* | 200 |
| 22 |  | ICU2/ Tap Water | *M. gadium* | >300 |
| 23 |  | Heart surgery1/ Tap water | *M. aurum* | >300 |
| 24 |  | Heart surgery2/ Tap water | *M. aurum* | 100 |
| 25 |  | Infectious1/ Tap water | *M. aurum* | >300 |
| 26 |  | Infectious2/ Tap water | *M. aurum* | >300 |
| 27 |  | Laboratory1/ Tap water | *M. mucogenicum* | 20 |
| 28 |  | Laboratory2/ Tap water | *M. mucogenicum* | 100 |
| 29 |  | Dentistry1/ Dental unit, water related to the syringe and turbine | *M. aurum* | >300 |
| 30 |  | Dentistry2/ Dental unit, water related to the syringe and turbine | *M. aurum* | >300 |
| 31 |  | Dentistry1/ Dental unit, water related to the syringe and turbine | *M. phocaicum* | 100 |
| 32 |  | Dentistry2/ Dental unit, water related to the syringe and turbine | *M. phocaicum* | 50 |
| 33 |  | Dentistry1/ Dental unit, water related to the syringe and turbine | *M. aurum* | >300 |
| 34 |  | Dentistry2/ Dental unit, water related to the syringe and turbine | *M. aurum* | >300 |
| 35 |  | Dentistry1/ Dental unit, water related to the syringe and turbine | *M. aurum* | >300 |
| 36 |  | Dentistry2/ Dental unit, water related to the syringe and turbine | *M. aurum* | >300 |
| 37 |  | Dentistry1/ Dental unit, water related to the syringe and turbine | *M. aurum* | >300 |
| 38 |  | Dentistry1/ Dental unit, water related to the syringe and turbine | *M. aurum* | >300 |
| 39 |  | Dentistry1/ Dental unit, water related to the syringe and turbine | *M. phocacicum* | 100 |
| 40 |  | Dentistry2/ Dental unit, water related to the syringe and turbine | *M. phocacicum* | 250 |
| 41 |  | Infectious1/ Tap water | *M. gordonae* | >300 |
| 42 |  | Infectious2/ Tap water | *M. gordonae* | >300 |
| 43 |  | Infectious1/ Tap water | *M. gordonae* | >300 |
| 44 |  | Infectious2/ Tap water | *M. gordonae* | >300 |
| 45 |  | Dialysis1 (Inlet water to the dialysis device) | *M. chelonae* | 50 |
| 46 |  | Dialysis2 (Inlet water to the dialysis device) | *M. chelonae* | 20 |
| 47 |  | Infectious1/ Tap water | *M. gordonae* | >300 |
| 48 |  | Infectious2/ Tap water | *M. gordonae* | >300 |
| 49 | No. 2 | ICU1/ Tap water | *M. phocaicum*  *M. gadium* | 50  >300 |
| 50 |  | ICU2/ Tap water | *M. phocaicum*  *M. gadium* | 100  200 |
| 51 |  | ICU1/ Tap water | *M. mucogenicum*  *M. aurum* | 20  >300 |
| 52 |  | ICU2/ Tap water | *M. mucogenicum*  *M. aurum* | 10  >300 |
| 53 |  | Women surgery1/ Tap water | *M. phocaicum* | 100 |
| 54 |  | Women surgery2/ Tap water | *M. phocaicum* | 250 |
| 55 |  | Women surgery1/ Tap water | *M. mucogenicum* | 200 |
| 56 |  | Women surgery2/ Tap water | *M. mucogenicum* | 50 |
| 57 |  | Men surgery1/ Tap water | *M. moriokaense* | 50 |
| 58 |  | Men surgery2/ Tap water | *M. moriokaense* | 10 |
| 59 |  | Men surgery1/ Tap water | *M. mucogenicum* | 100 |
| 60 |  | Men surgery2/ Tap water | *M. mucogenicum* | 50 |
| 61 |  | Heart surgery1/ Tap water | *M. phocaicum* | 100 |
| 62 |  | Heart surgery2/ Tap water | *M. phocaicum* | 150 |
| 63 |  | Heart surgery1/ Tap water | *M. phocaicum*  *M. aurum* | 50  >300 |
| 64 |  | Heart surgery2/ Tap water | *M. phocaicum*  *M. aurum* | 50  >300 |
| 65 |  | Dialysis1 (Inlet water to the dialysis device) | *M. mucogenicum* | 200 |
| 66 |  | Dialysis2 (Inlet water to the dialysis device) | *M. mucogenicum* | 100 |
| 67 |  | Dialysis1 (Inlet water to the dialysis device) | *M. phocaicum* | 10 |
| 68 |  | Dialysis2 (Inlet water to the dialysis device) | *M. phocaicum* | 150 |
| 69 |  | Infectious1/ Tap water | *M. mucogenicum* | >300 |
| 70 |  | Infectious2/ Tap water | *M. mucogenicum* | >300 |
| 71 |  | Infectious1/ Tap water | *M. gordonae* | >300 |
| 72 |  | Infectious2/ Tap water | *M. gordonae* | >300 |
| 73 |  | Infectious1/ Tap water | *M. gordonae*  *M. novocastrense*  *M. mucogenicum* | >300  >300  >300 |
| 74 |  | Infectious2/ Tap water | *M. gordonae*  *M. novocastrense*  *M. mucogenicum* | >300  >300  >300 |
| 75 | No. 3 | Laboratory1/ Tap water | *M. gordonae* | >300 |
| 76 |  | Laboratory2/ Tap water | *M. gordonae* | >300 |
| 77 |  | Operating room1/ Tap water | *M. mucogenicum* | 200 |
| 78 |  | Operating room2/ Tap water | *M. mucogenicum* | 100 |
| 79 |  | Operating room1/ Tap water | *M. lentiflavum* | 20 |
| 80 |  | Operating room2/ Tap water | *M. lentiflavum* | 10 |
| 81 |  | ICU1/ Tap water | *M. lentiflavum* | 5 |
| 82 |  | ICU2/ Tap water | *M. lentiflavum* | 30 |
| 83 |  | ICU1/ Tap water | *M. lentiflavum* | 10 |
| 84 |  | ICU2/ Tap water | *M. lentiflavum* | 15 |
| 85 | No. 4 | Dialysis1 (Inlet water to the dialysis device) | *M. lentiflavum* | 10 |
| 86 |  | Dialysis2 (Inlet water to the dialysis device) | *M. lentiflavum* | 10 |
| 87 |  | Dialysis1 (Inlet water to the dialysis device) | *M. gordonae* | 1 |
| 88 |  | Dialysis2 (Inlet water to the dialysis device) | *M. gordonae* | 100 |
| 89 |  | Men internal1/ Tap water | *M. gordonae* | >300 |
| 90 |  | Men internal2/ Tap water | *M. gordonae* | >300 |
| 91 | No. 5 | Operating room1/ Tap water | *M. gordonae* | 50 |
| 92 |  | Operating room2/ Tap water | *M. gordonae* | >300 |
| 93 |  | Emergency1/ Tap water | *M. lentiflavum* | 50 |
| 94 |  | Emergency2/ Tap water | *M. lentiflavum* | 10 |
| 95 |  | Emergency1/ Tap water | *M. lentiflavum*  *M. florentinum* | 100  10 |
| 96 |  | Emergency2/ Tap water | *M. lentiflavum*  *M. florentinum* | 50  10 |
| 97 |  | Infectious1/ Tap water | *M. florentinum* | >300 |
| 98 |  | Infectious2/ Tap water | *M. florentinum* | >300 |
| 99 | No. 6 | Emergency1/ Tap water | *M. gadium* | 50 |
| 100 |  | Emergency2/ Tap water | *M. gadium* | >300 |
| 101 |  | ICU1/ Tap water | *M. gadium* | >300 |
| 102 |  | ICU2/ Tap water | *M. gadium* | >300 |
| 103 |  | ICU1/ Tap water | *M. gordonae* | >300 |
| 104 |  | ICU2/ Tap water | *M. gordonae* | >300 |
| 105 |  | Heart surgery1/ HCD | *M. chelonae* | 50 |
| 106 |  | Heart surgery2/ HCD | *M. chelonae* | 10 |
| 107 |  | Heart surgery1/ Tap water | *M. aurum* | >300 |
| 108 |  | Heart surgery2/ Tap water | *M. aurum* | >300 |
| 109 |  | CCU1/ Tap water | *M. gadium* | >300 |
| 110 |  | CCU2/ Tap water | *M. gadium* | 100 |
| 111 |  | CCU1/ Tap water | *M. gadium* | >300 |
| 112 |  | CCU2/ Tap water | *M. gadium* | >300 |

| **Row** | **Hospital number** | **Sampling location** | **Isolated strain- Incubated at 37°C** | **CFU 500 ml^-1^** |
| --- | --- | --- | --- | --- |
| 1 | No. 1 | Dentistry1/ Dental unit, water related to the syringe and turbine | *M. porcinum* | >300 |
| 2 |  | Dentistry2/ Dental unit, water related to the syringe and turbine | *M. porcinum* | >300 |
| 3 |  | Dentistry1/ Dental unit, water related to the syringe and turbine | *M. porcinum* | >300 |
| 4 |  | Dentistry2/ Dental unit, water related to the syringe and turbine | *M. porcinum* | >300 |
| 5 |  | Women internal1/ Tap water | *M. kansasii* | >300 |
| 6 |  | Women internal2/ Tap water | *M. kansasii* | >300 |
| 7 |  | Women internal1/ Tap water | *M. kansasii* | 150 |
| 8 |  | Women internal2/ Tap water | *M. kansasii* | >300 |
| 9 |  | ICU1/ Tap water | *M. kansasii* | 100 |
| 10 |  | ICU2/ Tap water | *M. kansasii* | >300 |
| 11 |  | Women surgery1/ Tap water | *M. kansasii* | >300 |
| 12 |  | Women surgery2/ Tap water | *M. kansasii* | >300 |
| 13 |  | Women surgery1/ Tap water | *M. kansasii* | >300 |
| 14 |  | Women surgery2/ Tap water | *M. kansasii* | 200 |
| 15 |  | Laboratory1/ Tap water | *M. mucogenicum* | 50 |
| 16 |  | Laboratory2/ Tap water | *M. mucogenicum* | 200 |
| 17 |  | Dentistry1 (Dental unit, water related to the syringe and turbine) | *M. porcinum* | >300 |
| 18 |  | Dentistry2 (Dental unit, water related to the syringe and turbine) | *M. porcinum* | 200 |
| 19 | No. 2 | Women surgery1/ Tap water | *M. fortuitum* | >300 |
| 20 |  | Women surgery2/ Tap water | *M. fortuitum* | >300 |
| 21 |  | Heart surgery1/ Tap water | *M. porcinum* | >300 |
| 22 |  | Heart surgery2/ Tap water | *M. porcinum* | >300 |
| 23 |  | Heart surgery1/ Tap water | *M. fortuitum* | >300 |
| 24 |  | Heart surgery2/ Tap water | *M. fortuitum* | >300 |
| 25 |  | Infectious1/ Tap water | *M. simiae* | >300 |
| 26 |  | Infectious2/ Tap water | *M. simiae* | >300 |
| 27 | No. 3 | Heart surgery1/ Tap water | *M. simiae* | >300 |
| 28 |  | Heart surgery2/ Tap water | *M. simiae* | >300 |
| 29 |  | Operating room1/ Tap water | *M. kansasii* | >300 |
| 30 |  | Operating room2/ Tap water | *M. kansasii* | >300 |
| 31 |  | Men surgery1/ Tap water | *M. simiae* | >300 |
| 32 |  | Men surgery2/ Tap water | *M. simiae* | >300 |
| 33 | No. 4 | Heart surgery1/ Tap water | *M. mucogenicum* | 100 |
| 34 |  | Heart surgery2/ Tap water | *M. mucogenicum* | 50 |
| 35 |  | Operating room1/ Tap water | *M. phocaicum* | 100 |
| 36 |  | Operating room2/ Tap water | *M. phocaicum* | >300 |
| 37 |  | ICU1/ Tap water | *M. kansasii* | >300 |
| 38 |  | ICU2/ Tap water | *M. kansasii* | >300 |
| 39 |  | Infectious1/ Tap water | *M. kansasii* | >300 |
| 40 |  | Infectious2/ Tap water | *M. kansasii* | >300 |
| 41 |  | Infectious1/ Tap water | *M. simiae* | >300 |
| 42 |  | Infectious2/ Tap water | *M. simiae* | >300 |
| 43 |  | Dialysis1 (Inlet water to the dialysis device) | *M. simiae* | >300 |
| 44 |  | Dialysis2 (Inlet water to the dialysis device) | *M. simiae* | >300 |
| 45 |  | Men surgery1/ Tap water | *M. simiae* | 100 |
| 46 |  | Men surgery2/ Tap water | *M. simiae* | >300 |
| 47 | No. 5 | ICU1/ Tap water | *M. simiae* | >300 |
| 48 |  | ICU2/ Tap water | *M. simiae* | >300 |
| 49 |  | Infectious1/ Tap water | *M. simiae* | >300 |
| 50 |  | Infectious2/ Tap water | *M. simiae* | >300 |
| 51 |  | Infectious1/ Tap water | *M. fortuitum* | >300 |
| 52 |  | Infectious2/ Tap water | *M. fortuitum* | >300 |
| 53 |  | Infectious1/ Tap water | *M. fortuitum* | >300 |
| 54 |  | Infectious2/ Tap water | *M. fortuitum* | >300 |
| 55 | No. 6 | Infectious1/ Tap water | *M. fortuitum* | >300 |
| 56 |  | Infectious2/ Tap water | *M. fortuitum* | >300 |
| 57 |  | Infectious1/ Tap water | *M. simiae* | >300 |
| 58 |  | Infectious2/ Tap water | *M. simiae* | >300 |
| 59 |  | Heart surgery1/ Tap water | *M. fortuitum* | >300 |
| 60 |  | Heart surgery2/ Tap water | *M. fortuitum* | >300 |
| 61 |  | Women surgery1/ Tap water | *M. kansasii* | 100 |
| 62 |  | Women surgery2/ Tap water | *M. kansasii* | 50 |
